# Brassinin Inhibits Cell Survival and Autophagy via Modulation of ROS Production and the AMPKα‐beclin1 Signaling Pathway in Gliomas

**DOI:** 10.1002/ptr.70420

**Published:** 2026-08-31

**Authors:** Sunil Rauniyar, Yuhui Wang, Qiaoshan Zhang, Shicheng Zhang, Jiale Yin, Tong Zhang, Shangfeng Gao, Rutong Yu

**Affiliations:** ^1^ Department of Neurosurgery The Affiliated Hospital of Xuzhou Medical University Xuzhou China; ^2^ Institute of Nervous System Diseases Xuzhou Medical University Xuzhou China

**Keywords:** AMPK, autophagy, brassinin, glioma, ROS

## Abstract

Brassinin, a phytoalexin compound derived mainly from Chinese cabbage, shows anticancer activity against several types of cancer. In this study, we aimed to investigate the role of brassinin in glioma progression and reveal the underlying mechanism. CCK8 assays were used to investigate the dose‐ and time‐dependent effects of brassinin on the growth of different glioma cell lines, and then colony formation assays were employed to measure cell proliferation. Next, we used flow cytometry/Western blotting to assess cell cycle progression, apoptosis, and reactive oxygen species (ROS) production, and employed Western blotting/fluorescence staining for LC3, p62, and other signaling molecules to evaluate autophagy. Finally, we confirmed the in vitro findings in both the subcutaneous and intracranial glioma models using hematoxylin and eosin staining, immunohistochemistry, and immunofluorescence. Brassinin blocked cell cycle progression, inhibited proliferation, and promoted apoptosis in glioma cells. Brassinin inhibited autophagy, induced ROS overproduction, and suppressed the expression/activity of AMPKα‐beclin1 signaling. The effect of brassinin on cell growth and survival was partially reversed by an ROS scavenger or an autophagy activator in glioma cells. Brassinin regulated the AMPKα/beclin1 signaling and autophagy via promoting ROS production in glioma cells. The anticancer role of brassinin was confirmed in subcutaneous and intracranial glioma models, and brassinin administration prolonged the survival of tumor‐bearing mice. Brassinin inhibited cell survival and autophagy in glioma cells via modulation of ROS production and the AMPKα‐beclin1 signaling pathway. Our study identified a promising botanical drug for the treatment of gliomas.

## Graphical abstract

Brassinin, a phytoalexin derived primarily from Chinese cabbage, exhibits potent anticancer activity against glioblastoma (GBM). Mechanistically, brassinin treatment elevates intracellular ROS levels in GBM cells, triggering two parallel outcomes: (i) direct induction of apoptosis, and (ii) suppression of cytoprotective autophagy through downregulation of the AMPKα–Beclin1 signaling axis. Both effects are abrogated by the ROS scavenger NAC. Notably, the autophagy activator rapamycin (Rapa) reverses the brassinin‑induced autophagic inhibition, whereas the autophagy inhibitor 3‑methyladenine (3‑MA) further potentiates this effect. Collectively, the enhanced pro‑apoptotic action combined with the blockade of pro‑survival autophagy culminates in inhibited tumor growth and prolonged animal survival.

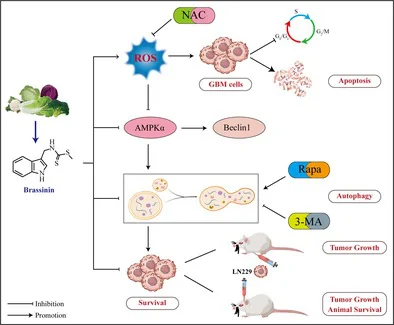

**Abbreviations**
3‐MA: 3‐methyladenine
AMPK: Adenosine monophosphate‐activated protein kinase
BCA: Bicinchoninic acid assay
CCK8: Cell counting kit‐8
DCFH‐DA: Dichlorofluorescein diacetate
DMEM: Dulbecco's modified Eagle's medium
DMSO: Dimethyl sulfoxide
ECL: Electrochemiluminescence
FITC: Fluorescein isothiocyanate
GBM: Glioblastoma multiforme
GFP: Green fluorescence protein
H&E: Hematoxylin and eosin
HA1800: Human astrocyte cell line
IC_50_
: Half‐maximal inhibitory concentration
IHC: Immunohistochemistry
LC3: Microtubule‐associated protein 1 light chain 3
Luc: Luciferase
NAC: *N*‐acetyl‐l‐cysteine
O.C.T: Optimal cutting temperature
PEG: Polyethylene glycol
PI: Propidium iodide
PVDF: Polyvinylidene fluoride
Rapa: Rapamycin
RFP: Red fluorescence protein
ROS: Reactive oxygen species
ULK1: Unc‐51 like autophagy activating kinase 1

## 1 Introduction

Glioblastoma multiforme (GBM) is the most lethal form of malignant glioma (Jackson et al. 2019). Despite recent advances in multimodal treatments, including maximum resection followed by radiotherapy and chemotherapy, the overall survival of patients with GBM remains only 15 months (Marenco‐Hillembrand et al. 2020; Grochans et al. 2022). Exploring new therapeutic strategies may improve the prognosis and quality of life of patients with GBM.

*N*‐(1‐H indol‐3‐ylmethyl)‐1‐methylsulfanyl‐methanethioamide (brassinin) is an indole phytoalexin present in cruciferous vegetables (e.g., Chinese cabbage). Brassinin plays an antiproliferative role in several types of cancer. In lung cancer, it can block cell cycle progression, induce apoptosis (Lee et al. 2015; Yang et al. 2019) in colon cancer, and enhance doxorubicin‐induced apoptosis (Park et al. 2021). Brassinin exerts chemopreventive and antitumorigenic effects in mouse models of breast cancer (Banerjee et al. 2008; Gu et al. 2022). However, studies revealing the role of brassinin in the proliferation and survival of glioma cells are lacking.

Macroautophagy (hereafter referred to as autophagy) is a defense mechanism in which cells use lysosomal pathways to eliminate defective organelles or molecules (Kroemer et al. 2010; Chen and White 2011). Adenosine monophosphate‐activated protein kinase (AMPK) and mammalian target of rapamycin (mTOR) are upstream signaling pathways of autophagy. They can directly phosphorylate Unc‐51 like autophagy activating kinase 1 (ULK1), thereby regulating downstream mediators (e.g., beclin1) to activate autophagy (Kim et al. 2011; Zhao and Klionsky 2011; Zhang et al. 2016). Rapamycin induces the early activation of the autophagy cascade via blockade of mTOR (Lin et al. 2018). Rapamycin enhances antitumor effects in glioma cells by upregulating autophagy (Wang et al. 2021). Interestingly, 3‐methyladenine (3‐MA), an inhibitor of early‐stage autophagy, also inhibits cancer cell survival (Wu et al. 2010; Sheng et al. 2013; Zhou et al. 2019). The inhibition of autophagy by 3‐MA increases cisplatin‐induced apoptosis in glioma cells (Zhang et al. 2015). Therefore, the targeting of autophagy is a promising therapeutic strategy for the treatment of GBM.

Dysregulation of reactive oxygen species (ROS) generation has been linked to the development, progression, and recurrence of tumors (Liou and Storz 2010; Ray et al. 2012; Yuan et al. 2015). Hong et al. reported that ROS overproduction caused by brassinin inhibits the proliferation and mediates apoptosis of liver cancer cells (Hong et al. 2021). In addition, inappropriate ROS generation regulates autophagy through AMPK‐mTOR signaling (Liou and Storz 2010; Wible and Bratton 2018). ROS inhibits autophagy via the ULK1 complex (Ci et al. 2014). Hence, we hypothesized that the brassinin‐mediated overproduction of ROS may inhibit autophagy. This inhibition might occur through the suppression of the AMPKα‐ULK1‐beclin1 pathway activity, consequently suppressing the survival of glioma cells.

In the current study, the effect of brassinin on the proliferation of different glioma cell lines was investigated using the Cell Counting Kit‐8 (CCK‐8) and colony formation assays. Flow cytometry was used to measure the effects of brassinin on cell cycle distribution, apoptosis, and ROS generation. Concurrently, the proteins related to cell cycle, apoptosis, autophagy, and activity of the AMPKα‐ULK1‐beclin1 pathway were detected by Western blotting. Finally, an ROS scavenger (*N*‐acetyl‐l‐cysteine (NAC)) was used to determine the requirement of ROS production for the regulation of autophagy and the anti‐glioma activity of brassinin. Subcutaneous and intracranial glioma models were used to study the effects of brassinin on the growth of glioma cells in vivo. This study aimed to elucidate the role and mechanism of brassinin in the malignant progression of gliomas.

## 2 Materials and Methods

### 2.1 Drugs and Antibodies

Brassinin (purity ≥ 98%, Chemical Abstracts Service (CAS) number: 105748‐59‐2) was purchased from LKT Laboratories (Minneapolis, MN, USA), and the chemical structure is provided in Figure 1A. A stock solution of brassinin (200 mM) was prepared in dimethyl sulfoxide (DMSO; MP Biomedicals, Santa Ana, CA, USA) and stored at −80°C. Different working concentrations were obtained by diluting stock solutions with Dulbecco's modified Eagle's medium (DMEM; KeyGEN BioTECH, Nanjing, China). NAC (CAS number: 616‐91‐1) was purchased from the Beyotime Institute of Biotechnology (Shanghai, China). Rapamycin (HY‐10219), 3‐MA (HY‐19312), and polyethylene glycol (PEG)‐300 (HY‐Y0873) were purchased from MedChem Express (Shanghai, China). We utilized the following primary antibodies for Western blotting detection: anti‐p21 (2947), anti‐p27 (3686), anti‐CDK4 (12790), anti‐CDK6 (3136), anti‐cyclin D1 (2922), anti‐PARP (9532), anti‐p53 (9282), anti‐Bax (2772), anti‐Bcl‐2 (5071), anti‐LC3 (3868), anti‐p62 (5114), anti‐phosphorylated (p)‐AMPKα (2535), anti‐AMPKα (2532), anti‐ULK1 (8054), and anti‐p‐ULK1 (37762). All reagents were purchased from Cell Signaling Technology (Danvers, MA, USA) and diluted 1:1000. Antibodies against CDK1 (1:1000; ab18) and cyclin B1 (1:2000; ab32053) were purchased from Abcam (Cambridge, UK). Beclin1 (1:1000; 11306‐1‐AP) was purchased from Proteintech (Wuhan, China). p‐Beclin1 (1:1000; AF2323) was obtained from Affinity (Zhenjiang, China). β‐Actin (1:1000; sc‐47778) was purchased from Santa Cruz Biotechnology (Santa Cruz, CA, USA).

**FIGURE 1:**
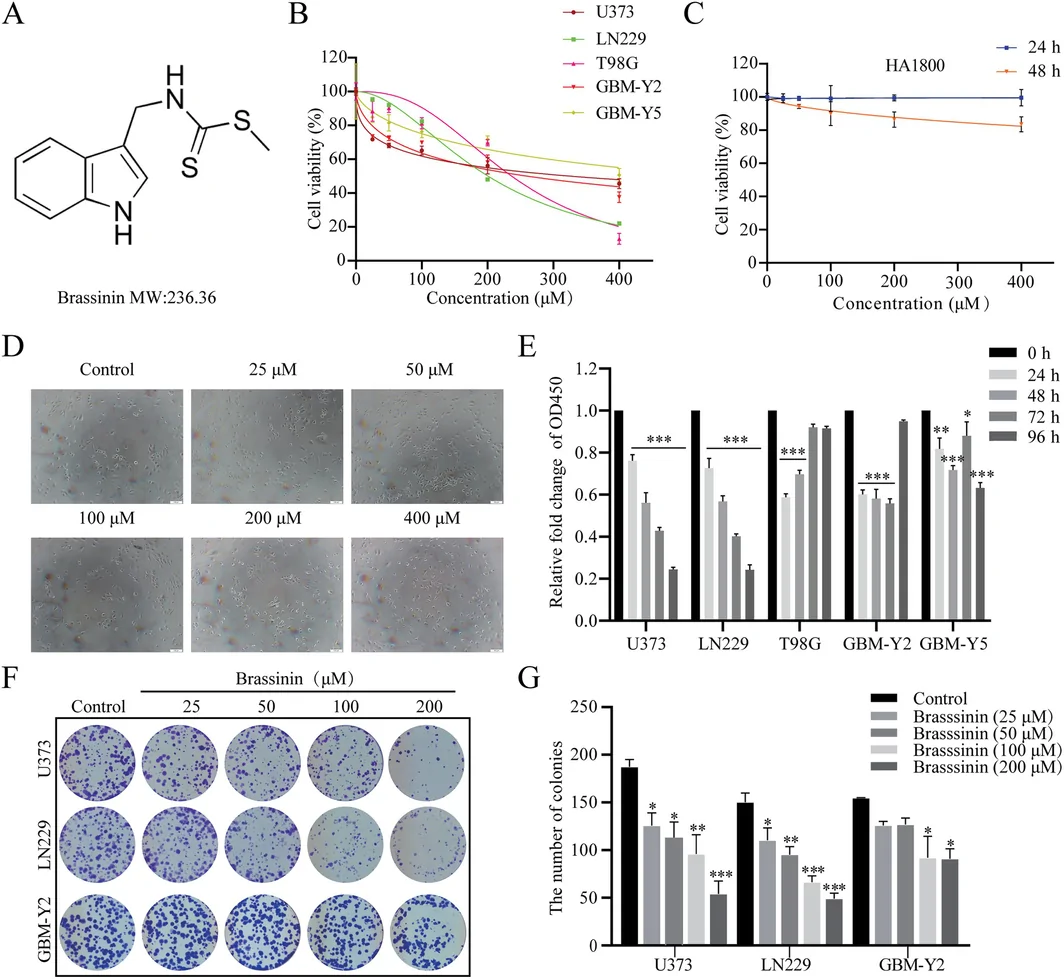
Brassinin inhibits the growth of human glioma cells. (A) Chemical structure of brassinin. (B) CCK8 assays were carried out to measure the effect of brassinin treatment on the viability of the indicated immortalized glioma cell lines. (C) CCK8 assay was performed to measure the effect of different concentrations and durations of brassinin treatment on the viability of human normal astrocytes (HA1800). (D) Morphological changes in HA1800 cells treated for 48 h with different doses of brassinin. Scale bar: 100 μm. (E) CCK8 assay showing the effects of different durations of brassinin treatment on the viability of the indicated immortalized glioma cell lines. (F, G) Colony formation assays were performed to evaluate the effect of brassinin on the proliferation of U373, LN229, and GBM‐Y2 cells. Representative images are shown in (F). Quantitative analyses of the number of colonies are shown in (G). *p < 0.05, **p < 0.01, ***p < 0.001.

### 2.2 Cell Culture

Human glioma cell lines (U373, LN229, and T98G) were purchased from the Shanghai Cell Bank of the Type Culture Collection of the Chinese Academy of Sciences (Shanghai, China). A human astrocyte cell line (HA1800) was a generous gift from Dr. Jiantong Jiao, as described in our previous study (Meng et al. 2021). Primary glioma cell lines (GBM‐Y2 and GBM‐Y5) were extracted from the surgical specimens of patients with glioma, as described previously (Gao et al. 2022). Cells were cultured in DMEM supplemented with 10% fetal bovine serum (Bio‐Channel Biotechnology, Nanjing, China) and incubated at 37°C in a humidified incubator in an atmosphere of 5% CO_2_.

### 2.3 Measurement of Cell Proliferation Using CCK8 Assays

A cell suspension (200 μL; 2000 cells/well) was seeded in 96‐well plates overnight. Then, cells were incubated with brassinin (0–400 μM) for 48 h. A CCK8 kit (Dojindo, Tokyo, Japan) was used to measure cell viability. Light‐field images were captured using a microscope (IX‐71; Olympus, Tokyo, Japan) to observe the effect of brassinin on the morphology of HA1800 cells. The half‐maximal inhibitory concentration (IC_50_) was calculated based on nonlinear regression analysis, as described in our recent study (Meng et al. 2021). To determine time‐dependent effects, cells were treated with brassinin (200 μM) and cell viability was measured at different time points (0–96 h) using CCK8 reagents.

### 2.4 Colony Formation Assay

At a density of 1000 cells/well, cells were plated in 6‐well plates and incubated overnight. Then, cells were exposed to brassinin (0–200 μM). After 48 h of incubation, the medium was changed, and the cells were allowed to grow for approximately 2 weeks. Cells were washed with phosphate‐buffered saline (PBS). After fixation with 4% paraformaldehyde for 20 min, the cells were washed with PBS. Next, the plates were stained with 0.5% crystal violet solution, and the colonies were photographed manually. ImageJ software (US National Institutes of Health, Bethesda, MD, USA) was used to quantify the number of colonies with > 50 cells per colony.

### 2.5 Cell Cycle Analyses

Briefly, 3 × 10^5^ cells were seeded in 6‐well plates and incubated overnight. After 48 h of treatment with brassinin (0, 50, 100, and 200 μM), cells were collected and fixed with 75% ethanol overnight at −20°C. Then, fixed cells were washed twice with PBS, stained with 500 μL of propidium iodide (PI) /RNase Staining Buffer (BD Biosciences, Franklin Lakes, NJ, USA) for 30 min, and analyzed by a flow cytometer (FACSCalibur; BD Biosciences).

### 2.6 Apoptosis

The cells (1 × 10^5^) were seeded in 6‐well plates and incubated overnight. Then, cells were incubated for 48 h with brassinin (0–200 μM). The FITC‐Annexin V Apoptosis Kit (Elabscience, Wuhan, China) was used to assess apoptosis. Briefly, cells in 100 μL of 1× Annexin V Binding Buffer were stained with 2.5 μL of annexin V‐fluorescein isothiocyanate (FITC) and 2.5 μL of propidium iodide (PI) at 20°C–25°C in the dark for 15–20 min. Then, 400 μL of 1× Annexin V Binding Working Solution was added to the tube and mixed gently. The percentage of apoptotic cells was determined using a flow cytometer (BD FACSCalibur).

### 2.7 ROS Determination

The fluorescent dye dichlorofluorescein diacetate (DCFH‐DA; Beyotime Institute of Biotechnology) was used to evaluate ROS production. Cells (5 × 10^5^) were plated in 6‐well plates and treated with brassinin (0–200 μM). After 48 h, the cells were collected and incubated in serum‐free medium containing DCFH‐DA (10 mM) for 30 min. The stained cells were washed three times and analyzed using a flow cytometer (BD FACSCalibur). The mean fluorescence intensity of DCFH‐DA and the percentage of ROS‐positive cells were calculated using FlowJo 10 software (Becton Dickinson, Ashland, OR, USA).

### 2.8 mRFP‐GFP‐Microtubule‐Associated Protein 1 Light Chain 3 (LC3) Puncta Assay

To observe autophagic flux, cells were transiently transfected with the mRFP‐GFP‐LC3 plasmid, as described in our previous study (Zhao et al. 2021). Twenty‐four hours after transfection, cells were treated with brassinin (100, 200 μM) for 48 h, rapamycin (500 nM) for 24 h, or NAC (7.5 mM) for 24 h. Next, cells were washed with PBS and fixed with 4% paraformaldehyde for 20 min. Cells were treated with 4′,6‐diamidino‐2‐phenylindole (DAPI) for 15 min to stain the nuclei. Images were acquired using an inverted microscope (IX71, Olympus).

### 2.9 Western Blotting

Cells (2.5 × 10^5^) were treated with brassinin (0, 50, 100, 200 μM). At the designated time points, the cells were lysed, and the protein concentration was determined using a BCA Protein Assay Kit (Beyotime Institute of Biotechnology) following the manufacturer's instructions. Equal amounts of protein were loaded onto 8%–15% polyacrylamide gels, separated by sodium dodecyl sulfate–polyacrylamide gel electrophoresis, and transferred to polyvinylidene difluoride (PVDF) membranes (GE Healthcare, Chicago, IL, USA). After being incubated with 3% bovine serum albumin to block any nonspecific binding, the PVDF membranes were probed with primary antibodies (at the dilutions stated in section 2.1) at 4°C overnight. The membranes were then incubated with horseradish peroxidase‐labeled goat anti‐rabbit/mouse IgG (1:4000; Abcam) for 2 h at room temperature. Signals were visualized using an ECL Substrate Kit (Thermo Fisher Scientific, Waltham, MA, USA) under the ChemiDoc Touch Imaging System (Bio‐Rad Laboratories, Hercules, CA, USA). ImageJ software was used to quantify band densities. The relative amount of each protein was calculated by normalizing the densitometric value of the protein to that of β‐actin.

### 2.10 Tumor Xenografts in Nude Mice

BALB/c nude mice (18–22 g) were purchased from the Laboratory Animal Center of Xuzhou Medical University. The animals were housed in a standard animal facility with free access to sterilized food and water. The protocol for the animal experiments was approved by the Experimental Animal Ethics Committee of Xuzhou Medical University (Approval No.: 202110A060, Xuzhou, China). We established subcutaneous and intracranial models of GBM using the LN229 cell line stably expressing mCherry and luciferase, which was used in our previous study (Meng et al. 2021).

For the subcutaneous model, cells (3 × 10^6^) were injected into the left flank of mice. When the tumor size reached approximately 40–100 mm^3^, the tumor‐bearing mice were randomly divided into Vehicle (*n* = 5) and Brassinin (*n* = 5) groups. Brassinin was dissolved in DMSO, and the drug solution was mixed with a solvent containing 25% PEG‐300, 0.5% Tween‐80, and 74.5% sterile H_2_O. Mice were treated with brassinin (75 mg/kg, i.p.) or an equal volume of the solvent for 2 weeks (5 days/week). The tumor size and body weight were recorded every 2 days. Tumor volume (calculated (in mm^3^) as (length × width^2^)/2) denoted tumor size. Xenograft tumors and primary organs were collected for hematoxylin and eosin (H&E) staining, immunohistochemistry (IHC), and immunofluorescence.

For the intracranial model, cells (1 × 10^6^ cells) were injected into the right striatum of nude mice. Tumor growth was monitored using a live imaging system (Berthold Technologies, Bad Wildbad, Germany). On day 9, the tumor‐bearing mice were divided into Vehicle and Brassinin groups (*n* = 12 for each group). The mice were treated with brassinin or solvent daily for 3 weeks. Some mice (*n* = 4 per group) were sacrificed on day 49, and the remaining mice were sacrificed when cachexia was observed.

### 2.11 Staining

Subcutaneously xenografted tumors and main organs (heart, kidneys, liver, spleen, and lungs) were collected, fixed in 4% paraformaldehyde, cryoprotected in 20% and 30% sucrose solutions, and embedded in O.C.T. compound. Sections were cut at a thickness of 10 μm with a cryostat microtome (Leica, Wetzlar, Germany). Intracranial xenografted tumors were collected, fixed in 10% formalin, embedded in paraffin, and sectioned at a thickness of 6 μm with a paraffin microtome (Leica).

IHC staining was performed as described previously (Zhang et al. 2018). A primary antibody against Ki67 (ZSJB‐Bio, Beijing, China) was used at a dilution of 1:100 for frozen sections and 1:200 for paraffin‐embedded sections. An ABC Elite kit (Vector Laboratories, Burlingame, CA, USA) was used to visualize primary antibodies. The sections were counterstained with hematoxylin (KeyGEN BioTECH) for 8–10 min. Images were captured using a microscope (DM2500; Leica).

Immunofluorescence staining was performed as described previously (Gao et al. 2021). We used the following primary antibodies: anti‐Bcl‐2 (1:400; 5071s; Cell Signaling Technology), anti‐p‐AMPKα (1:100; 2535; Cell Signaling Technology), and anti‐beclin1 (1:100; 11306‐1‐AP; Proteintech). Cell nuclei were counterstained with DAPI (1:1000) for 5 min. Immunofluorescent images were acquired using a microscope (IX71; Olympus, Tokyo, Japan).

### 2.12 Statistical Analyses

The in vitro experiments were repeated at least three times. Quantitative data are expressed as the mean ± SD. Prism 8.0 (GraphPad, La Jolla, CA, USA) was used for the statistical analyses. One‐way analysis of variance (ANOVA) followed by Dunnett's or Tukey's test was used to compare differences between more than two groups. Student's *t*‐test was used to compare two groups. *p* < 0.05 was considered significant.

## 3 Results

### 3.1 Brassinin Inhibits the Proliferation of Glioma Cells and Exhibits Low Toxicity to Human Normal Astrocytes

Different glioma cell lines were incubated with brassinin for 48 h. The CCK8 assay showed that brassinin administration resulted in a concentration‐dependent reduction in the viability of three immortalized cell lines (U373, T98G, and LN229) and two primary GBM cell lines (GBM‐Y2 and GBM‐Y5) (Figure 1B). In comparison, brassinin exhibited minimal effect on the viability of human astrocytes (HA1800) and showed no obvious changes in the number or morphology of cells with increasing drug concentration (Figure 1C,D). The IC_50_ values of brassinin against these different cell types are listed in Table 1. A time‐dependent inhibitory effect of brassinin on the viability of these glioma cell lines was observed (Figure 1E). However, cell viability was restored in certain cell lines (e.g., T98G and GBM‐Y2) at 72 and 96 h following brassinin treatment. Colony formation assays showed that brassinin treatment inhibited the proliferation of U373, LN229, and GBM‐Y2 cells (Figure 1F,G). These findings indicate that brassinin exhibited antiproliferative effects in glioma cells but had low toxicity in normal astrocytes.

**TABLE 1:** The IC_50_ values of Brassinin for glioma cell lines.

Cell lines	48 h IC_50_ (μM)	*R* ^2^
LN229	200.8 ± 1.29	0.9848
U373	325.6 ± 1.22	0.9683
T98G	236.7 ± 1.40	0.9048
GBM‐Y2	260.1 ± 4.84	0.9257
GBM‐Y5	547.5 ± 1.28	0.8134

### 3.2 Brassinin Blocks Cycle Progression in Glioma Cells

To determine whether brassinin treatment inhibited the proliferation of glioma cells by modulating the progression of the cell cycle, flow cytometry was used to detect the distribution of glioma cells in the cell cycle. Brassinin treatment (200 μM) for 48 h increased the percentage of cells in the G1 phase and decreased the percentage of cells in the S phase for U373 cells and GBM‐Y2 cells (Figure 2A,B). Western blotting analysis of G1‐checkpoint proteins revealed that the expression levels of p27, p21, and p53 increased in a concentration‐dependent manner, whereas the expression of cyclin D1, CDK4, and CDK6 decreased gradually in U373 and LN229 cells after brassinin treatment (Figure 2C,D). The two G2/M‐checkpoint proteins, CDK1 and cyclin B1, showed no concentration‐dependent decrease in response to brassinin treatment (Figure 2C,D). These findings suggest that brassinin suppressed the growth and proliferation of glioma cells by arresting the cell cycle in the G1 phase.

**FIGURE 2:**
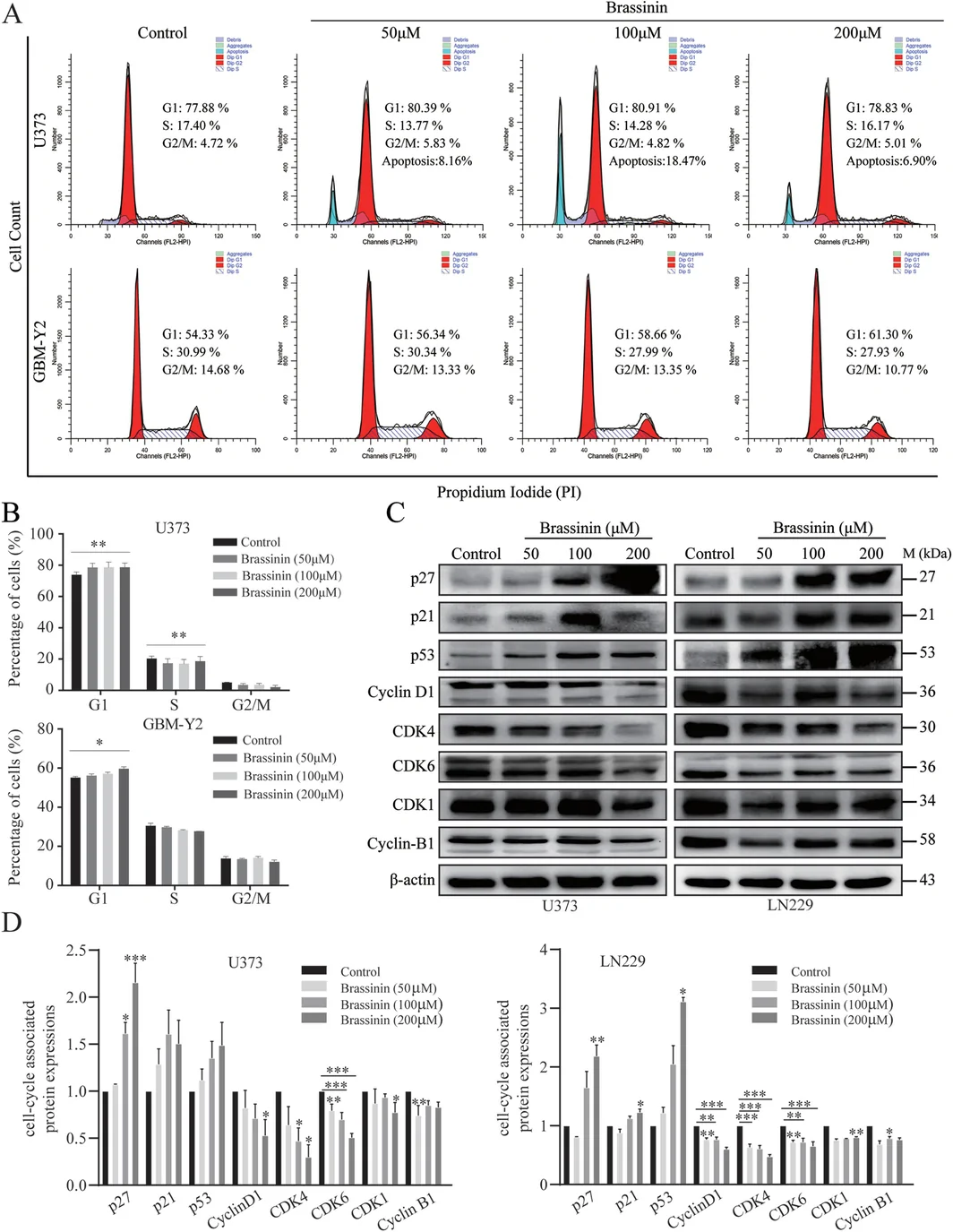
Brassinin blocks cycle progression in glioma cells. (A, B) Flow cytometry was used to analyze the effect of brassinin treatment on cell cycle progression in U373 cells and GBM‐Y2 cells. Representative histograms of flow cytometry data are shown in (A). The percentage of cells at G1, S, and G2 phases is shown in (B). (C, D) Western blotting analysis showing the expression changes in p27, p21, p53, cyclin D1, CDK4, CDK6, CDK1, and cyclin B1 following brassinin treatment at different concentrations. Representative blot images are shown in (C). Quantification Graphs are shown in (D). M: Molecular weight. *p < 0.05, **p < 0.01, ***p < 0.001.

### 3.3 Brassinin Promotes Apoptosis of Glioma Cells

Brassinin induced a marked increase in the percentage of U373 cells in the sub‐G1 phase (Figure 2A). Therefore, we investigated the role of brassinin in glioma cell apoptosis. Flow cytometry analysis showed that the percentage of apoptotic and sub‐G1‐phase cells increased with increasing brassinin concentration in U373 cells and LN229 cells (Figure 3A–C). Western blotting showed that brassinin treatment increased the expression of pro‐apoptotic proteins (Bax, PARP, and cleaved PARP) and decreased the expression of the anti‐apoptotic protein Bcl‐2 in U373 and LN229 cells in a dose‐dependent manner (Figure 3D). These findings indicate that brassinin inhibited the growth of glioma cells by triggering apoptosis.

**FIGURE 3:**
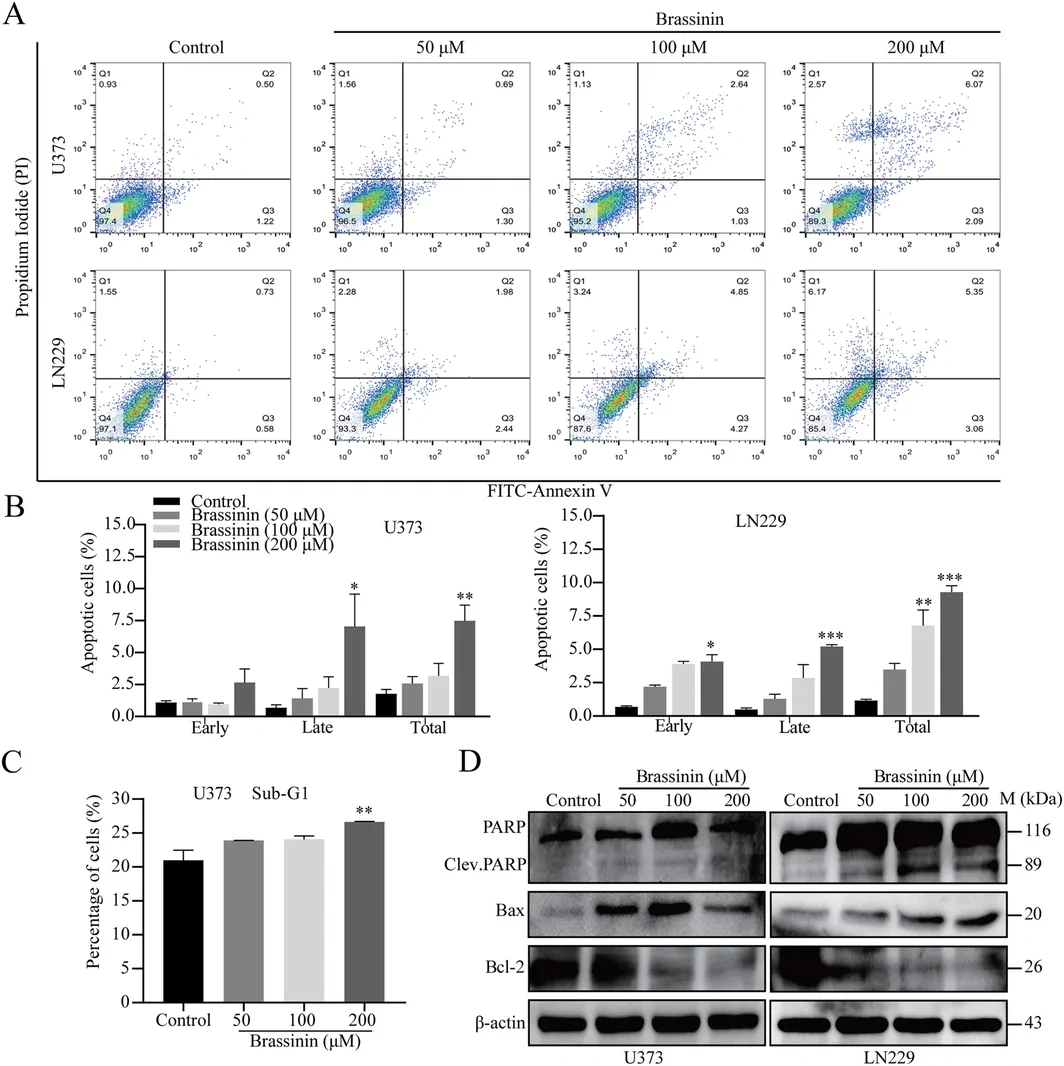
Brassinin promotes the apoptosis of glioma cells. (A, B) Flow cytometry was used to analyze the effect of Brassinin treatment on the apoptosis of U373 cells and LN229 cells. Representative histograms of flow cytometry data are shown in (A). The percentage of apoptotic cells is shown in (B). (C) The percentage of cells at the sub‐G1 phase according to flow cytometry is shown in Figure 2A. (D) Western blots showing the expression of cleaved PARP/PARP, Bax, and Bcl‐2 in U373 cells and LN229 cells following brassinin treatment. M: Molecular weight. *p < 0.05, **p < 0.01, ***p < 0.001.

### 3.4 Brassinin Inhibits the Survival of Glioma Cells by Promoting ROS Generation

Hong et al. found that brassinin treatment leads to ROS production in liver cancer cells (Hong et al. 2021). Therefore, we first measured ROS levels in glioma cells following brassinin treatment. Flow cytometry using DCFH‐DA fluorescence showed that the administration of brassinin led to a dose‐dependent increase in ROS generation in U373 and LN229 cells (Figure 4A,B). The ROS scavenger NAC inhibited the brassinin‐induced increase in ROS production caused by brassinin treatment (Figure 4C,D). CCK8 and colony formation assays revealed that brassinin‐mediated inhibition of cell proliferation was reversed (at least in part) by NAC in U373 cells and LN229 cells (Figure 4E–G). NAC rescued the brassinin‐induced reduction in the levels of CDK4/6 and Bcl‐2, and increased the levels of p53, cleaved PARP, and Bax in U373 cells (Figures 4H,I and S1). These results indicate that brassinin inhibited glioma cell survival by promoting ROS generation.

**FIGURE 4:**
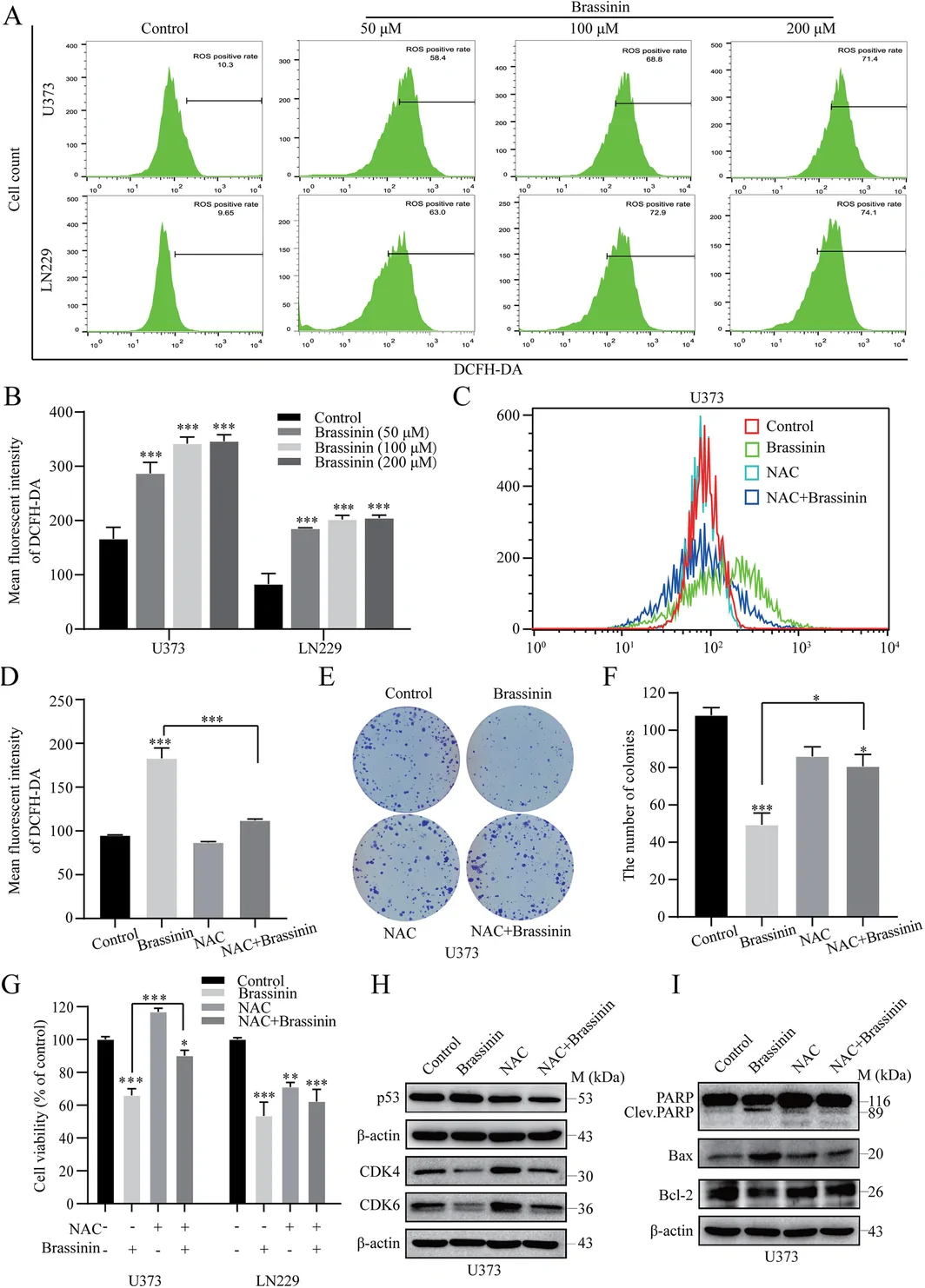
Brassinin inhibits survival by inducing ROS generation in glioma cells. (A, B) Flow cytometry data of the DCFH‐DA fluorescent probe was used to measure the effect of brassinin treatment on ROS levels in U373 and LN229 cells. Representative histograms of flow cytometry data are shown in (A). Mean fluorescent intensity of DCFH‐DA is shown in (B). (C–I) U373 cells were pre‐incubated with NAC (7.5 mM) for 24 h, followed by treatment with brassinin (100 μM) for an additional 48 h. Flow cytometry of the DCFH‐DA fluorescent probe was used to analyze the ROS level. Representative histograms of flow‐cytometry data are shown in (C). Mean fluorescent intensity of DCFH‐DA is shown in (D). Assays (colony formation and CCK8) were used to measure the proliferation of U373 cells and LN229 cells. Representative colony images are shown in (E), quantitative graphs are shown in (F) and (G). Western blotting was used to measure expression of proteins related to the cell cycle (H) and apoptosis (I). M: Molecular weight. *p < 0.05, **p < 0.01, ***p < 0.001.

### 3.5 Brassinin Inhibits the Survival of Glioma Cells by Regulating Autophagy

Inappropriate ROS generation can regulate autophagy in glioma cells (Dong et al. 2023; Wible and Bratton 2018). Therefore, we investigated whether the administration of brassinin influenced autophagy in these cells. Western blotting analysis showed that treatment with brassinin reduced the protein expression of LC3‐II (an autophagy marker) and increased the protein expression of p62 (a marker of autophagy degradation) in U373 and LN229 cells (Figure 5A,B), indicating the inhibition of autophagy. Next, we measured the activity of the upstream signaling pathway that regulates autophagy. The expression and/or activity levels of AMPKα, ULK1, and beclin1 decreased in a concentration‐dependent manner in both U373 cells and LN229 cells (Figure 5C). These data demonstrate that brassinin inhibited autophagosome formation in glioma cells.

**FIGURE 5:**
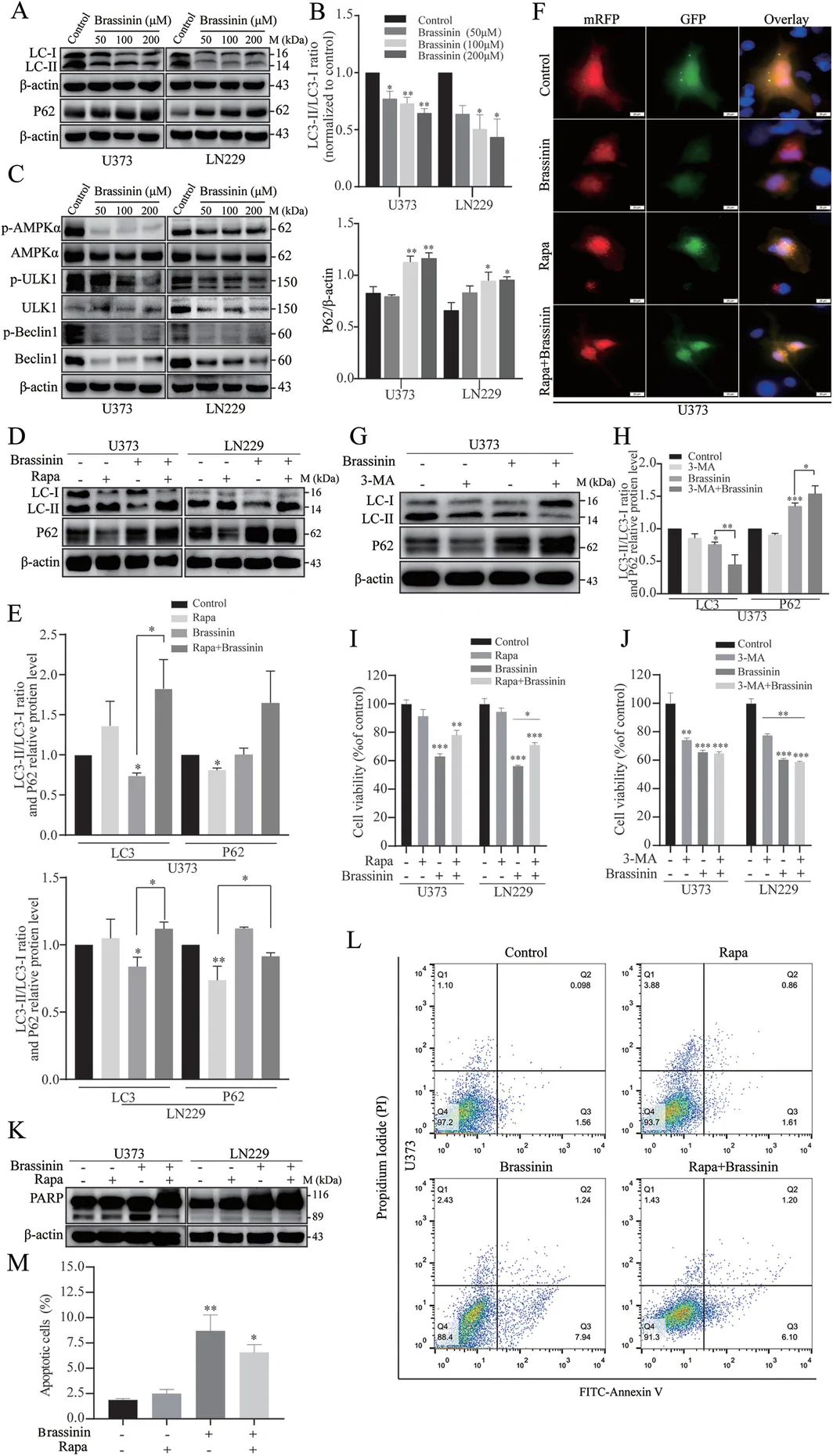
Brassinin inhibits proliferation and promotes apoptosis by regulating autophagy in glioma cells. (A–C) Western blots showing changes in levels of autophagy markers (A) and autophagy‐associated signaling molecules (C) in glioma cells following brassinin treatment. Graphs showing changes in protein expression of LC3 and p62 are shown in (B). (D–J) Rapamycin (Rapa) or 3‐methyladenine (3‐MA) was added to glioma cells 24 h before brassinin treatment. Next, cells were treated with or without brassinin for 48 h. Representative Western blots of LC3 and p62 are shown in (D) (Rapa) and (G) (3‐MA). Quantification graphs showing the changes in protein expression of LC3 and p62 are shown in (E) (Rapa) and (H) (3‐MA). Fluorescent microscopy observation of LC3 puncta is shown in (F). Scale bar: 20 μm. CCK8 assay measurement of cell viability is shown in (I) (Rapa) and (J) (3‐MA). (K–M) Glioma cells were pre‐incubated with Rapa for 24 h, followed by brassinin treatment for 48 h. Western blots of cleaved PARP/PARP are shown in (K). Flow cytometry was used to assess the apoptosis of U373 cells. Representative histograms of flow cytometry data are shown in (L). The percentage of apoptotic cells is shown in (M). The concentrations used for the rescue experiment were 200 μM for brassinin, 500 nM for rapamycin, and 5 mM for 3‐MA. *p < 0.05, **p < 0.01; ***p < 0.001.

We next explored the effect of co‐administration of rapamycin (an autophagy activator) and brassinin on autophagy in glioma cells. The loss of LC3‐II caused by brassinin treatment was markedly reversed by the co‐administration of rapamycin and brassinin to U373 and LN229 cells (Figure 5D,E). Similarly, the rapamycin‐induced decrease in p62 expression was rescued by this combination treatment (Figure 5D,E). We further examined autophagy flux by transiently transfecting glioma cells with the mRFP‐GFP‐LC3 plasmid. After 48 h of exposure to brassinin, the number of LC3‐positive puncta was significantly reduced in U373 cells compared to that in untreated cells. However, this change disappeared when rapamycin was used to initiate autophagy (Figure 5F). More importantly, combined treatment with 3‐MA (an inhibitor of early autophagy) and brassinin showed a synergistic effect on autophagy inhibition, as evidenced by decreased LC3‐II and increased p62 levels (Figure 5G,H).

Next, we determined whether rapamycin could rescue the brassinin‐induced inhibition of glioma cell survival. CCK8 assay results showed that the reduced viability caused by brassinin was significantly rescued in the presence of rapamycin in U373 cells and LN229 cells (Figure 5I). Furthermore, combined treatment with 3‐MA and brassinin synergistically inhibited cell growth, particularly of LN229 cells (Figure 5J). Flow cytometry and Western blotting of cleaved PARP demonstrated that brassinin‐induced apoptosis was partially reversed by rapamycin treatment in U373 and LN229 cells (Figures 5K–M and S1). Hence, brassinin appears to inhibit the growth and promote the apoptosis of glioma cells by inhibiting autophagy.

### 3.6 Brassinin Suppresses AMPKα‐beclin1 Signal Transduction and Autophagy by Inducing ROS Overproduction in Glioma Cells

An increase in ROS levels has been postulated to inhibit autophagy by suppressing the expression of the autophagy‐related protein, ULK1 (Ci et al. 2014). We explored whether the inhibitory effect of brassinin on the AMPKα‐ULK1‐beclin1 signaling pathway and autophagy was mediated by increased ROS levels in glioma cells. Consistent with the results shown in Figure 5, brassinin treatment led to a decrease in LC3‐II levels and an increase in p62 levels, which were reversed by the addition of NAC to U373 and LN229 cells (Figure 6A,B). Transfection of the mRFP‐GFP‐LC3 plasmid into LN229 cells revealed that brassinin treatment decreased the number of LC3‐positive puncta, which was counteracted by combined administration of NAC and brassinin (Figure 6C). Co‐administration with NAC reversed the brassinin‐mediated reductions in p‐AMPKα and beclin1 levels, but not p‐ULK1 levels, in U373 and LN229 cells (Figure 6D). These results suggest that brassinin negatively regulated AMPKα‐beclin1‐mediated autophagy by promoting cellular ROS production.

**FIGURE 6:**
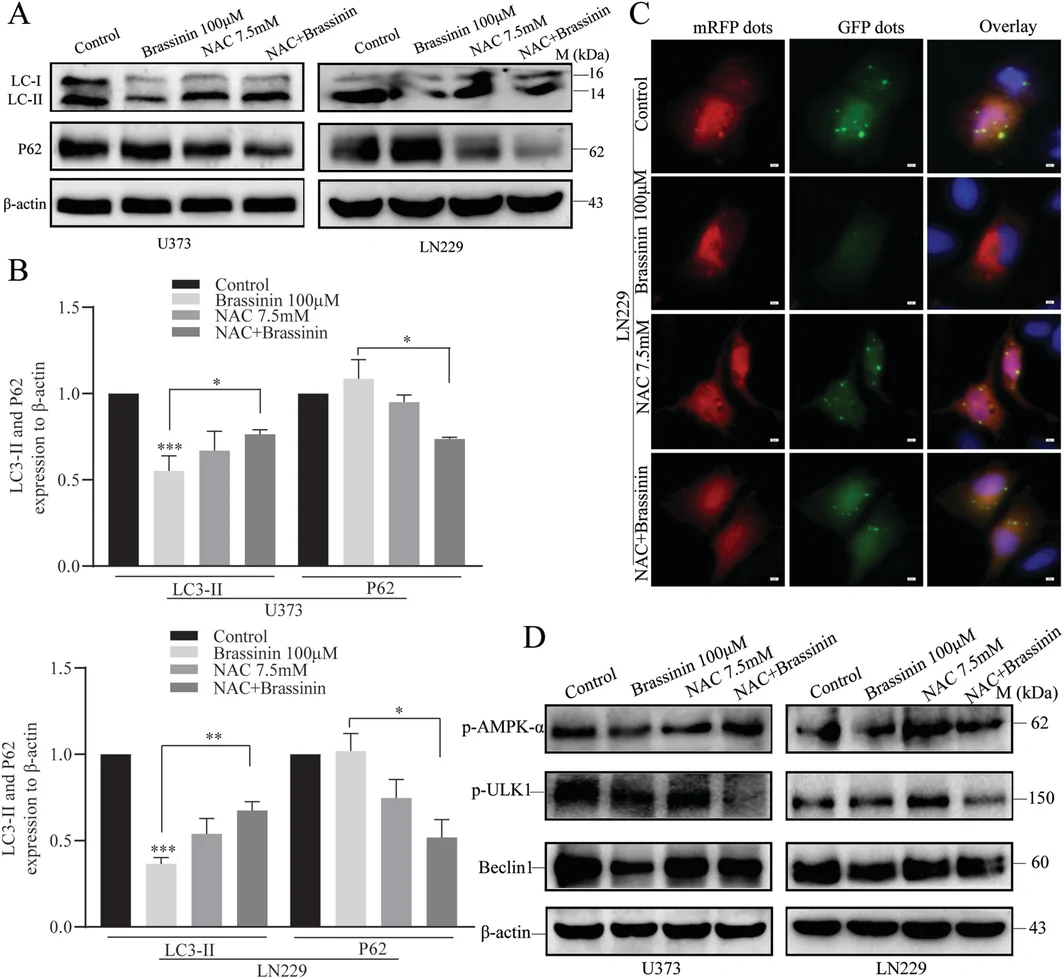
Brassinin negatively regulates the AMPKα‐beclin1 signaling pathway by increasing ROS generation in glioma cells. Glioma cells were pre‐incubated with NAC (7.5 mM) for 24 h, and then treated or not treated with brassinin (100 μM) for another 48 h. Western blots of LC3 and p62 are shown in (A). Quantification graphs showing the changes in protein expression of LC3‐II and p62 are shown in (B). Fluorescent microscopic observation of LC3 puncta is shown in (C). Scale bar: 20 μm. Western blots of phosphorylated AMPKα (p‐AMPKα), ULK1, and beclin1 are shown in (D). M: Molecular weight. *p < 0.05, **p < 0.01, ***p < 0.001.

### 3.7 Brassinin Inhibits Glioma Growth in a Subcutaneous Xenograft Model

We established a subcutaneous xenograft model using LN229 cells stably expressing mCherry and luciferase, and brassinin was administered intraperitoneally (i.p.). In the Brassinin group, the reduction in tumor volume became evident from day 8 onwards, and the reduction in tumor weight was significant on day 14 in comparison to the Vehicle group (Figure 7A–C). Brassinin treatment had no obvious effect on the body weight of tumor‐bearing mice (Figure 7D), and no abnormalities were observed in histological examinations of the heart, spleen, liver, lungs, and kidneys (Figure 7E). IHC analysis in xenografted gliomas revealed a significant reduction in the number of Ki67‐positive cells after brassinin treatment (Figure 7F,G), and immunofluorescent staining showed that the signals of Bcl‐2, p‐AMPKα, and beclin1 were markedly reduced compared with those in the Vehicle group (Figure 7H). These findings suggest that brassinin suppressed proliferation, promoted apoptosis, and inhibited the activity of autophagy signaling pathways in gliomas with good safety and tolerability.

**FIGURE 7:**
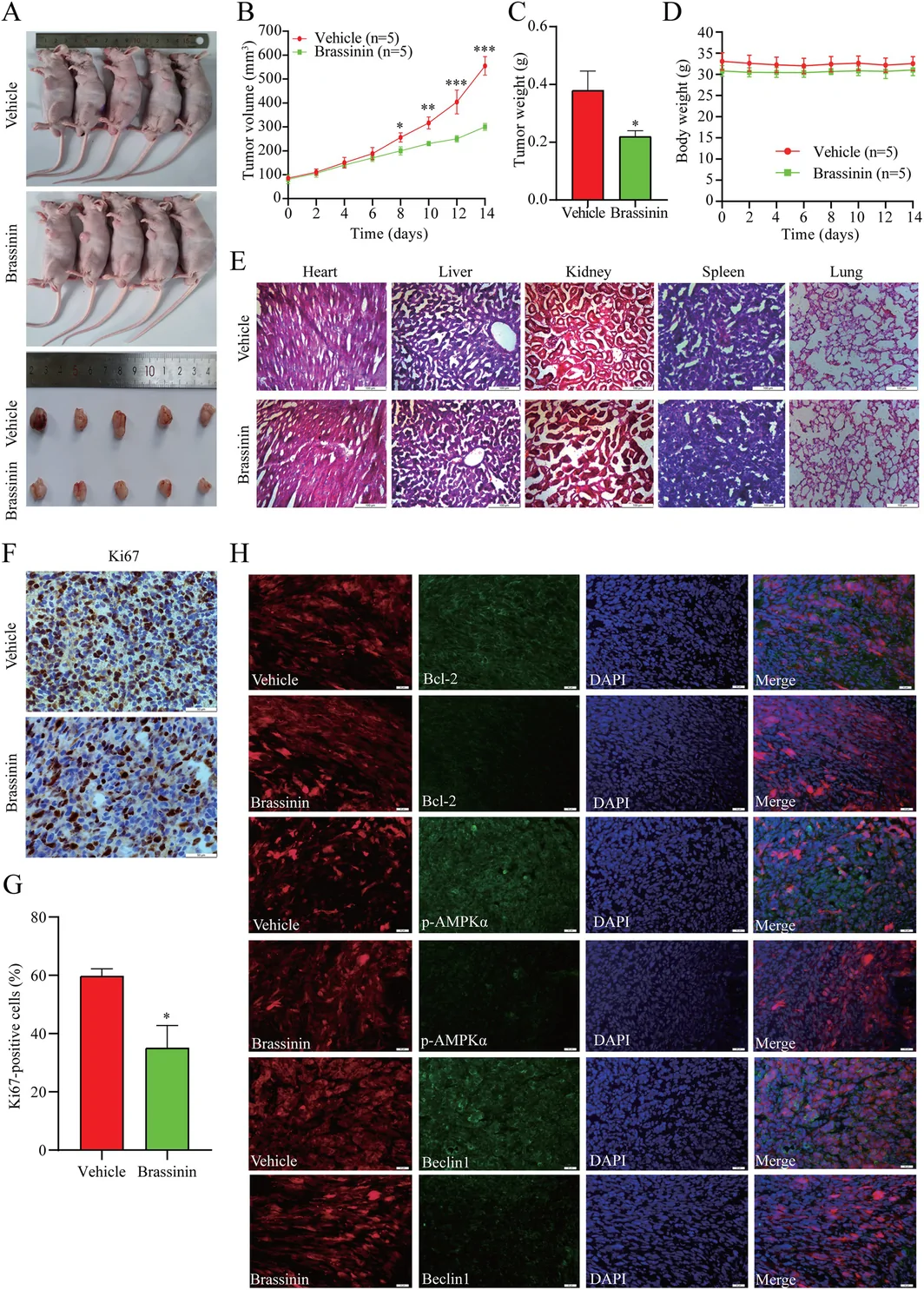
Brassinin inhibits the growth of glioma in a subcutaneous xenograft model. LN229 cells expressing mCherry were used to establish a subcutaneous glioma model, and then brassinin was administered (i.p.). (A) Xenograft tumors. (B) Changes in tumor volume. (C) Changes in tumor weight. (D) Bodyweight changes. (E) H&E staining showed the effect of brassinin on the morphology of the heart, liver, kidneys, spleen, and lungs. Scale bar: 100 μm. (F, G) IHC analysis of Ki67‐positive staining showed the effect of brassinin on the proliferation of tumor cells. Representative Ki67‐positive images are shown in (F). The percentage of Ki67‐positive cells is shown in (G). Scale bar: 50 μm. (H) Immunofluorescent staining revealed the changes in the immunoreactivity of Bcl‐2, p‐AMPKα, and beclin1 in xenograft tumor sections. Scale bar: 100 μm. *p < 0.05, **p < 0.01, ***p < 0.001.

### 3.8 Brassinin Inhibits Glioma Growth and Extends the Survival of Animals in an Intracranial Xenograft Model

An intracranial xenograft model was established using the abovementioned LN229 cells. Brassinin was administered (i.p.), and tumor growth was monitored (Figure 8A). The xenografted gliomas in the mouse brains were relatively smaller in the Brassinin group than in the Vehicle group, as reflected by bioluminescence imaging in living animals (Figure 8B). H&E staining further confirmed that tumor size was significantly reduced in the Brassinin group (Figure 8C). IHC analysis of Ki67 staining in brain sections showed an inhibitory effect of brassinin on tumor proliferation in vivo (Figure 8D). Brassinin treatment extended the median survival of tumor‐bearing mice from 29.5 days to 33.5 days (Figure 8E). These data suggest that brassinin inhibited the growth of gliomas in vivo and prolonged the survival of tumor‐bearing mice.

**FIGURE 8:**
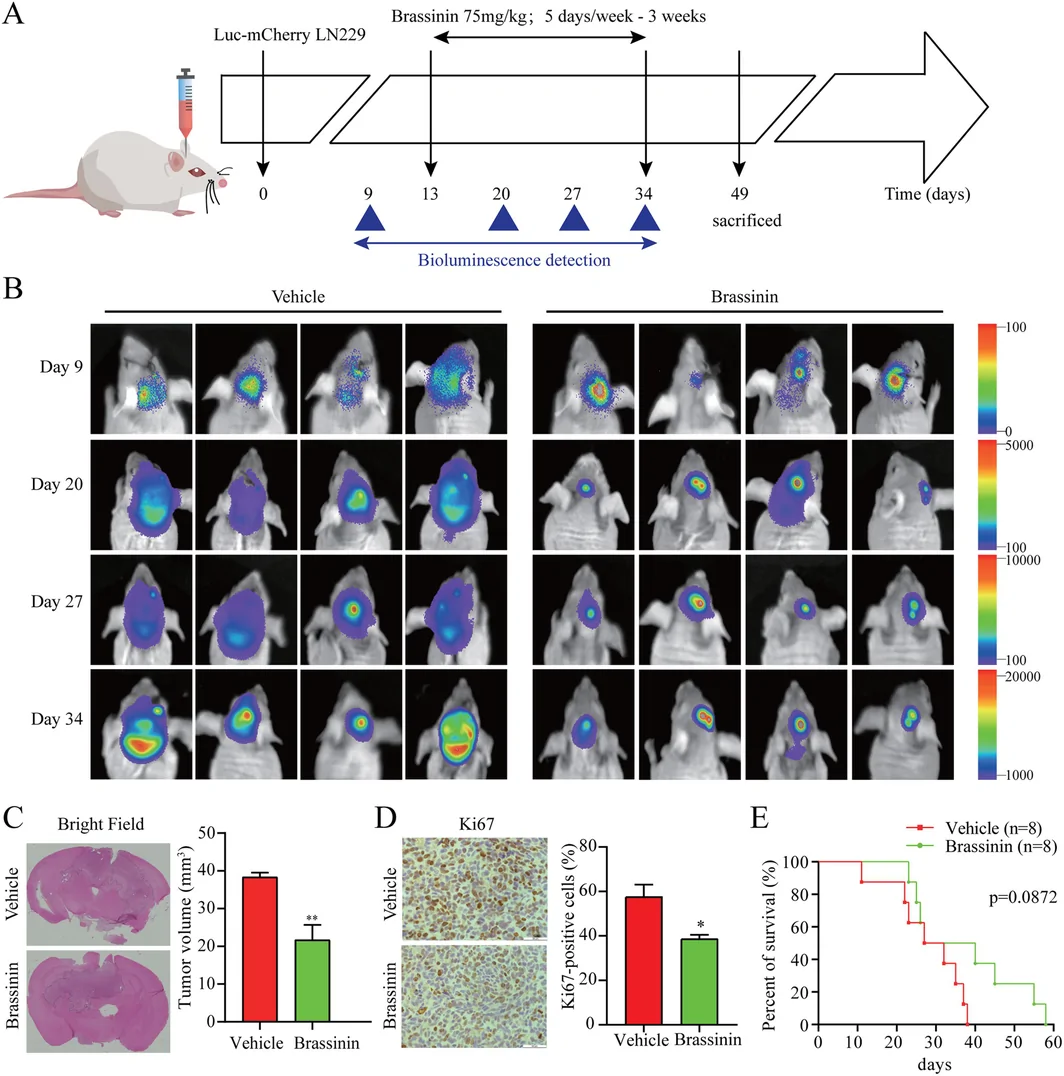
Brassinin inhibits the growth of gliomas and extends the survival of tumor‐bearing mice. LN229 cells expressing luciferase (Luc) and mCherry were used to establish a model of intracranial glioma, and then brassinin was administered (i.p.). (A) Protocol used for tumor implantation and drug administration. (B) Bioluminescent imaging was used to reflect the changes in glioma growth in living animals. (C) H&E staining showing the volume changes of intracranial tumors between the Brassinin and Vehicle groups. (D) IHC analysis of Ki67 staining was used to show the effect of brassinin treatment on the proliferation of tumor cells. Scale bar: 50 μm. (E) Kaplan–Meier analysis was employed to reveal the effect of brassinin on the survival of tumor‐bearing mice. *p < 0.05, **p < 0.01.

## 4 Discussion

Brassinin is a phytoalexin that has sparked great interest owing to its roles in several types of cancer (Mezencev et al. 2003; Feitelson et al. 2015; Chripkova et al. 2016). We found that brassinin blocked cell cycle progression, triggered apoptosis, and inhibited the proliferation of glioma cells. More importantly, brassinin inhibited AMPKα‐beclin1‐mediated autophagy and promoted apoptosis by increasing ROS generation. The anti‐glioma effect of brassinin has been verified in both subcutaneous and intracranial GBM models.

Phytoalexins, such as brassinin and resveratrol, mediate their anticancer effects by triggering cell cycle arrest and promoting apoptosis in cancers of the lung, colon, and liver, as well as malignant natural killer cells (e.g., leukemia, lymphomas) (Izutani et al. 2012; Quoc Trung et al. 2013; Lee et al. 2015; Hong et al. 2021). In accordance with these findings, we showed that brassinin blocked cell cycle progression and increased the levels of p21, p53, and p27, while decreasing the levels of cyclin D1, CDK4, and CDK6 in glioma cells. Brassinin treatment upregulated the expression of pro‐apoptotic proteins (Bax, PARP, and cleaved PARP) and downregulated the expression of the anti‐apoptotic protein Bcl‐2 in glioma cells; the latter was confirmed in glioma xenograft tissues. Therefore, brassinin may inhibit the growth of glioma cells by preventing cell cycle progression and promoting apoptosis, which can be generalized to other types of cancer cells.

Protective autophagy promotes the degradation of damaged organelles and proteins, which are necessary for cell survival (Chavez‐Dominguez et al. 2020). 3‐MA (an inhibitor of early autophagy) plays an antiproliferative role in glioma cells (Liu et al. 2019). We found that brassinin inhibited autophagy and induced apoptosis in glioma cells and xenografts. Similar to other phytoalexins, such as resveratrol (Lin et al. 2012), brassinin blocked autophagy at an early stage, as reflected by decreased LC3‐II levels and increased p62 levels in glioma cells. This observation was substantiated by the reduced activity of the AMPKα‐beclin1 signaling pathway in brassinin‐treated cells. Moreover, brassinin in combination with 3‐MA had a synergistic effect on autophagy inhibition and cell survival, whereas rapamycin (an autophagy activator) rescued the inhibitory effect of brassinin on autophagy and cell growth in glioma cells. These results demonstrate that brassinin inhibited early autophagy to impede the survival of glioma cells. However, we did not perform scanning electron microscopy analysis, which limits the strength of our conclusions.

Typically, antitumor agents trigger an increase in ROS levels in glioma cells, leading to cell death (Ma et al. 2016; Rinaldi et al. 2016). The overproduction of ROS reduces protective autophagy and induces apoptosis in leukemia cells (Ci et al. 2014). Brassinin is an antitumor agent that promotes ROS generation and inhibits autophagy and the survival of glioma cells. The ROS scavenger NAC reversed the suppressive effects of brassinin on glioma cell survival and autophagy. Taken together, these findings suggest that brassinin‐mediated ROS production was required for the inhibition of autophagy and survival of glioma cells. ROS generation is an upstream event of AMPK signaling (Yi et al. 2013), and the AMPKα‐beclin1 pathway is a master regulatory pathway that drives autophagy (Zhang et al. 2016; Liu et al. 2020). We showed that brassinin reduced the activity of the AMPKα‐beclin1 pathway, which could be reversed by the ROS scavenger NAC. These data indicate that brassinin acts through the ROS/AMPKα‐beclin1 signaling pathway to inhibit autophagy and induce the apoptosis of glioma cells.

The present study has some limitations that must be addressed. First, there may be signaling molecules or pathways other than the AMPKα‐beclin1 signaling that contribute to the inhibitory effect of brassinin on autophagy and cell survival; thus, further studies are required. Second, temozolomide induces protective autophagy, which drives glioma treatment resistance (Yan et al. 2016). However, we did not evaluate the combined effect of brassinin and temozolomide on glioma growth, which warrants further investigation. Third, although brassinin has antitumor effects in several types of cancer (Mehta et al. 1995; Mezencev et al. 2003), the direct target of this drug is currently unclear. Resolving this issue will undoubtedly stimulate the clinical application of brassinin in patients with cancer. Therefore, in future studies, we aim to design experiments carefully to address these issues.

## 5 Conclusions

Brassinin inhibited the survival of glioma cells, which was accompanied by ROS overproduction, reductions in the activity of the AMPKα‐beclin1 pathway, and inhibition of autophagy. The ROS/AMPKα‐beclin1 signaling was required for brassinin‐induced inhibition of autophagy and cell survival. Brassinin inhibited tumor growth and prolonged the survival of animals in glioma models. These findings demonstrate the anti‐glioma role of brassinin and reveal its possible mechanism of action, thereby providing new options for glioma drug therapy.

## Author Contributions

**Sunil Rauniyar:** data curation, formal analysis, investigation, software, writing – original draft. **Yuhui Wang:** investigation, methodology, software. **Qiaoshan Zhang:** formal analysis, investigation, visualization. **Shicheng Zhang:** formal analysis, software. **Jiale Yin:** methodology. **Tong Zhang:** funding acquisition, resources. **Shangfeng Gao:** conceptualization, funding acquisition, writing – review and editing. **Rutong Yu:** funding acquisition, project administration, resources.

## Funding

This work was supported by the National Natural Science Foundation of China (82002632, 82072763, 82473218), Advanced Program of the Affiliated Hospital of Xuzhou Medical University (PYJH2024206), High‐Level Hospital Construction Project of Jiangsu Province (2023601001), and The Key Research & Development Plan of Xuzhou City (KC22103).

## Conflicts of Interest

The authors declare no conflicts of interest.

## Supporting information


**Figure S1:** (A) U373 cells were pre‐incubated with NAC (7.5 mM) for 24 h, and then treated with Brassinin (100 μM) for another 48 h. Western blot was used to analyze the expression of proteins related to apoptosis. (B) Glioma cells were pre‐incubated with Rapa (500 nM) for 24 h followed by Brassinin (200 μM) treatment for another 48 h. Western blot was used to analyze the cleaved PARP/PARP levels in both U373 and LN229 cells. M: Molecular weight.

## Data Availability

Data will be made available on request.
