# Supplementary material for: Brassinin Inhibits Cell Survival and Autophagy via Modulation of ROS Production and the AMPKα‐beclin1 Signaling Pathway in Gliomas
Source: Phytother Res. 2026 Aug 31;40(11):6233–48. doi: 10.1002/ptr.70420 (PMC13753666; doi:10.1002/ptr.70420)
Supplement: Supplementary file 1 [file PTR-40-6233-s001.docx]

**Supplementary Figure 1**

(**A**) U373 cells were pre-incubated with NAC (7.5 mM) for 24 h, and then treated with Brassinin (100 μM) for another 48 h. Western blot was used to analyze the expression of proteins related to apoptosis. (**B**) Glioma cells were pre-incubated with Rapa (500 nM) for 24 h followed by Brassinin (200 μM) treatment for another 48 h. Western blot was used to analyze the cleaved PARP/PARP levels in both U373 and LN229 cells. M: Molecular weight.
